# RespiraConNosotros: A Viable Home-Based Telerehabilitation System for Respiratory Patients

**DOI:** 10.3390/s21103318

**Published:** 2021-05-11

**Authors:** Beatriz María Bermejo-Gil, Fátima Pérez-Robledo, Rocío Llamas-Ramos, Luís Augusto Silva, André Sales-Mendes, Valderi Reis Quietinho Leithardt, Inés Llamas-Ramos

**Affiliations:** 1 Department of Nursery and Physiotherapy, Faculty of Nursing and Physiotherapy, University of Salamanca, 37008 Salamanca, Spain; beatriz.bermejo@usal.es (B.M.B.-G.); fatima_pr@usal.es (F.P.-R.); inesllamas@usal.es (I.L.-R.); 2 Expert Systems and Applications Lab (ESALAB), Faculty of Science, University of Salamanca, 37008 Salamanca, Spain; andremendes@usal.es; 3 COPELABS, University Lusófona—ULHT, 1749-024 Lisbon, Portugal; 4 VALORIZA, Research Center for Endogenous Resources Valorization, Institute Polytechnic of Portalegre, 7300-555 Portalegre, Portugal; valderi@ipportalegre.pt

**Keywords:** COVID-19, home therapy, pulmonary, respiratory exercises, SARS-CoV-2, telerehabilitation

## Abstract

Currently, there are more than 1.55 million cases of SARS-CoV-2 infection in Spain. Of these, it is estimated that around 45% will present respiratory complications, which represents approximately 620,000 patients who will need respiratory rehabilitation. The health system has no resources for this huge quantity of patients after the hospital discharge to finish the complete recovery and avoid the chronicity of the symptoms. We propose an application named RespiraConNosotros. The application has been created and designed to guide users in performing respiratory rehabilitation exercises, especially for COVID-19 patients, and it also facilitates patient–physiotherapist contact via chat or video calling to help patients. It is accessible for all users and on all devices. All exercises would be guided and supervised by a specialized physiotherapist who suggests, adapts, and guides the exercise according to the function level of each patient. Data obtained was satisfactory; all patients pointed out the easy access, the intuitive format, and the advantage of communicating with an expert. Concerning functional assessment, all participants improved their score on the Borg scale after performing the intervention with the application.This platform would help respiratory patients to make rehabilitation treatments to recover their pulmonary function and to decrease or eliminate the possible complications they have. It never substitutes any prescribed treatment. In conclusion, RespiraConNosotros is a simple, viable, and safe alternative for the improvement and maintenance of respiratory capacity and patient’s functionality affected by COVID-19. It could be used as a complement to face-to-face treatment when the situation allows it.

## 1. Introduction

Telerehabilitation has been a highly researched new treatment modality in recent years. This option consists of the provision of rehabilitation services through telecommunications networks or the Internet offering remote treatments [1]. Since SARS-CoV-2 emerged and caused the collapse of health systems in 2020, people are not able to receive their face-to-face treatments, chronic patients are unable to continue them, and professionals could not attend all of the consultations. The new situation caused by this virus has accelerated the implementation of telerehabilitation in various systems of health care, from medical consultations to the performance of rehabilitation treatments in musculoskeletal pathologies [2].

Concerning this new modality of treatment, new technologies have been incorporated into our daily lives through applications that allow us to register different aspects of our everyday life and have been demonstrated to be effective for promoting the general population’s health [3]. In recent years, there have been many mobile applications that try to promote users’ health with the aim of giving feedback on their health status and providing health information. They were focused especially on physical activity and diet-like “SoSu-life” application. This application was related with personalized feedback, practical tips, and tricks to reduce weight in specific groups of employees within workplace settings. Other applications related to lifestyle improvement and health-promoting behaviors such as “Solar Cell” for sun protection, an application to control vitamin D and calcium intake, and applications related to improving bone health, among others, have been investigated [3]. The most widely used applications are those related to physical activity and weight control. These applications monitor the user activity as well as their changes in habits, giving feedback on the individual’s health status. However, this application has not been tested in people with pathologies [3,4].

Several studies have focused on applications related to alcohol and smoking; other studies have detailed applicaitons for pathologies such as asthma, breastfeeding, cancer, depression, diabetes, headaches, heart disease, HIV, hypertension, iron deficiency/anemia, low vision, and pain. Studies have also focused on applications to control obesity, weight management, physical activity, mindfulness, general health and fitness, and women’s health. All of these applications have been used to improve health [5]. Physicians point out the advantages of e-health. In general, professionals with experience are more open to appreciating the benefits and advantages of this technology, which outweigh the difficulties, although they ask for training to improve their technology skills [6]. Mobile applications and computer programs have been used for different purposes, such as disease prevention, diagnosis or treatment [7,8,9].

Furthermore, the scientific literature has explored the effectiveness of these treatments in different chronic pathologies as diabetes mellitus, chronic lung disease, and cardiovascular disease with interventions which range from 3 months to 1 year [10]. One of the fields in which applications have been used is respiratory conditions, such as chronic obstructive pulmonary disease (COPD) [11] or cystic fibrosis [12]. All the applications used have demonstrated their effectiveness, although some authors highlighted the need for more research to determine the real effects that they have on pathologies [13].

Considering this new technology’s application, the idea of applying it in the current situation caused by the pandemic arises. Currently, there are more than 1.55 million cases of SARS-CoV-2 infection in Spain [14]. Of these, it is estimated that around 45% will present respiratory complications [15], which represents approximately 620,000 patients who will need respiratory rehabilitation. Once these patients are discharged from hospitals, they did not have rehabilitation programs due to clinical collapse and the impossibility to guarantee the safest conditions for their implementation.

Faced with this health emergency, a change in the treatment protocols and follow-ups carried out on these patients is necessary. Some authors propose mobile applications to fill resource deficits and access a larger population in this uncontrolled situation. They highlighted the urgent need for applications to monitor and control the patient’s symptoms or patients’ health in general, who are clustered at home and also in intensive unit care to obtain as much as possible information about the most people [16].

As it has already been mentioned, one of the main sequelae is respiratory involvement. Patients who have overcome COVID-19, the SARS-CoV-2 disease, often report dyspnea or shortness of breath, a sensation that is reflected in alterations of respiratory capacity or function [17]. A correct physiotherapy treatment focused on pulmonary training and pulmonary function restoration will improve these sequelae; however, its face-to-face rehabilitation is a high risk. Despite this, the ratio of patients needing rehabilitation has increased the need for a viable and safe alternative treatment. In this contextual framework, the use of new technologies for the patient’s treatment with the respiratory sequela is proposed as an alternative, since they have been used for other symptom management with effective results [18]. This solution would respond to outpatient treatment, but it could also improve early rehabilitation, which is necessary in the treatment of this disease. Authors support the importance of early pulmonary rehabilitation during the hospital admission and even 6 to 8 weeks after discharge [19].

The study reason was to propose a therapeutic alternative to help respiratory rehabilitation and patient’s functional recovery. The proposal was a newly implemented platform that responds to current needs. The platform was designed by experts: a doctor in physiotherapy, a nurse, and computer engineers. It was a powerful and complete research group. The health-related members found and researched the rehabilitation needs of these patients, as well as the need for remote assistance. Furthermore, physiotherapists selected the best exercises for rehabilitation and performed the video guide. Computer engineers were able to identify to develop the needed connection between patients and caregivers throughout this easy and intuitive platform.

In this study, we aimed to evaluate the viability of the platform as well as present its development. Regarding viability, health-related results were evaluated on voluntary participants with dyspnea and fatigue complaints after COVID-19.

With regard to patient data privacy, GDPR addresses the need to protect sensitive data and the inevitable risk of data theft. Encryption reinforces that all sensitive information must be covered by an acceptable level of security at both its source and destination [8]. According to Ibraimi et al. [20], patient confidentiality is one of the significant obstacles in obtaining medical data, as some information is not shared for fear of being stored in databases that do not comply with security regulations. The HIPAA Privacy Rule [21] deals with the security of sensitive patient information in the medical field. It is a United States federal law created in 1996 to impose standards to protect such information and prevent its sharing without the patient’s consent.

The target of this project is to contribute to health improvements, through an application with respiratory exercises and physiotherapists follow-up, to improve the rehabilitation of patients who have suffered from COVID-19 disease, achieving cardiorespiratory improvements that increase their functional level. One of the main advantages is offered by its applicability both in the hospital environment and at home. Furthermore, it also facilitates communication between patients and health care providers.

The rest of the paper is organized as follows. In Section 2, we introduce the methodology for an application, enhancing the telerehabilitation exercises and the proposed application. In Section 3, we show the results and the evaluation. In Section 4 and Section 5, we present the discussion about the application and conclusion, respectively.

## 2. Methodology

In this section, we present the proposed system. The first consideration to be taken when developing collaborative applications where users can have different roles with responsibilities, in this case clinical, within the application, is that the skills must be validated by an administrator user. To explain the methodology used to develop this application, we will start by explaining how the respiratory rehabilitation exercises were chosen and how the technologies, methods, and mechanisms for the design and implementation of the collaborative platform were used.

### 2.1. Respiratory Exercises

The method chosen to guide the user in carrying out the exercises has been by use of video. This method is extensively used for teaching because it allows having the same information in a redundant way such as audio instructions, visual instructions, or subtitles. Apart from this, the use of video as a means to guide the user to perform the exercises allows the user to perform the exercise more successfully because he has to try to repeat only the exercises that are already at the specified time indications.

The videos included in the application present different breathing exercises to work and strengthen the respiratory muscles and increase ventilation. These exercises are designed following the main action guidelines for patients with different respiratory pathologies from acute conditions such as COVID-19 to chronic conditions such as COPD [22]. These guidelines determine that therapy through respiratory exercises is effective and highly recommended, which justifies the implementation of ventilatory training programs. In all of them, respiratory exercise programs that start easily and progress are established. For this reason, it was decided to divide the exercises and establish an order for the patients to progressively increase the difficulty of their training.

Usually, the workouts begin with the simple learning of the breathing rhythm, in which air is taken in through the nose and exhaled through the mouth [23,24]. The RespiraConNosotros application also includes this section. The first exercises are based on becoming aware of the body’s breathing, learning an adequate rhythm and differentiating between coastal and diaphragmatic breathing, both important in ventilator mechanics. The ventilation rhythm is set by the physiotherapist who takes the video, with inspiration pauses of 2–3 s to achieve a thoracic expansion (fibrosis). The physiotherapist insists on postural control—specifically, on maintaining the best possible position and paying special attention to the exercise performed—to integrate the movement and automate it. In addition, they are asked to place their hands on the chest or abdomen to get feedback on how they are performing the exercise. When they do it correctly, they will feel how these areas swell with the entry of air and they will understand that the exercises are well done [24].

The position of the exercises varies; it is not advisable to do them always in the same position, but the ideal is to do them lying down, sitting, and standing [25]. The RespiraConNosotros application has several videos in each of these positions.

The next level of difficulty is established by adding the movement of the limbs while taking the breaths. It is more difficult because it requires a great coordination between the respiratory and arms movements, but it is a notable benefit, because it more greatly affectsthe mobilization and flexibilization of the limbs. In many respiratory diseases, thoracic movements are usually restricted due to lack of mobility, deformation, or the appearance of stiffness [26]. Because of this, it is necessary to flex the rib cage and maintain mobility of the upper limbs.

For this, different exercises are proposed in all the positions mentioned above (lying down, sitting, and standing) in which they are asked to move their arms while breathing. These movements, as suggested by the treatment guidelines for respiratory patients, occur according to this principle: the opening movements (spread arms, trunk tilt, shoulders flexion) occur during inspiration, and during expiration, there is return to rest or initial position [22].

Again, it is pointed constantly in controlling posture and awareness of the movement performed. The errors that occur most frequently are explained in the video to prevent them from occurring in patients who view the activity.

As the training progresses, resistance exercises are also added. They are indicated for different respiratory diseases [22] and can be performed with weights or elastic bands. In the videos presented, they are proposed with elastic bands, although they could be perfectly performed with dumbbells in the hands instead of the elastic band. These exercises are intended to enhance the respiratory muscles, give it strength and functionality. It is necessary to have previously carried out the previous exercises to avoid alterations in the movement or compensations when making the effort. The majority of the proposed exercises were performed without resistance previously; patients already know them and know what the dynamics of movement are like. This helps that when it comes to performing them against resistance, they are performed with the same accuracy. The rest of the videos are focused once again on postural control, attention to exercise, and possible errors that may appear are explained to prevent patients from commenting on them.

Finally, functional exercises have also been designed. These exercises imply that the patient recognizes them, they are habitual for him or her, and this makes it easier for him oir her to perform them correctly. Other action guidelines also include exercises of this type [27]. In our case, drinking straws and balloons have been used as materials. Balloon exercises have been placed at the end of training, since they require breathing force and overcome the resistance offered by the material to be inflated. These exercises are effective and motivate patients more, so they are also applied to avoid problems of lack of adherence.

These exercises have great health benefits for respiratory patients because they reduce the severity of symptoms, medication relief, and emergency room visits [23]. For this reason, they have been selected for this respiratory rehabilitation application program.

### 2.2. Application

This platform is available for all devices and has been designed for patients who need rehabilitation of the respiratory system but cannot access face-to-face treatment due to the collapse of clinical resources, people who have been forced to quarantine, or people who are not feeling safe outside home. Although the objective has been to explain, teach, and guide users in the performance of respiratory rehabilitation exercises, especially for COVID patients, the application can be applied in different areas of rehabilitation where users have to perform daily exercises for the recovery of any type of physical injury. It also facilitates patient–physiotherapist contact every time they need using devices that both already have through message or video calling. It should be noted that this application does not replace any prescribed treatment; it is a complement.

**Figure 1:**
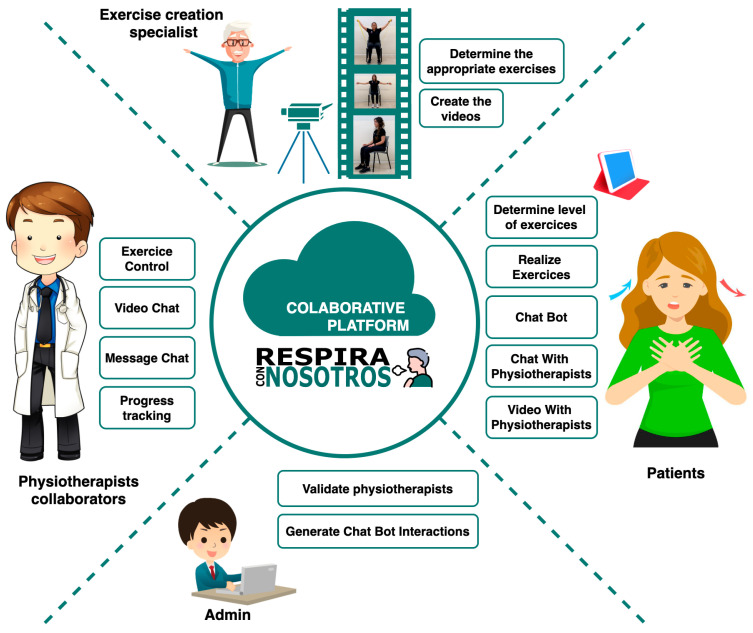
Collaborative platform roles.

Figure 1 can show the different roles of the RespiraConNosotros collaborative platform. From the beginning, this platform has been designed in a collaborative way between different departments of the University of Salamanca such as the Department of Nursery and Physiotherapy and the department of Informatics. It has also been designed to be scalable by collaboratively increasing the number of physiotherapists to assist patients that join the application. In the figure, we can see the roles of admin and exercise creation specialist that would be at an initial point would be permanently fixed. The administrator users would be responsible for validating that the physiotherapists meet the required requirements to be able to attend to the patients, in addition to generating the interactions of the chatbot with the patients of the application. Users with the role of exercise creation specialist are responsible for studying which exercises are appropriate to incorporate into the application and carry out the creation of the audiovisual content and its subtitles on the platform. There are possibilities of sending notifications and alerts directed according to the application’s criteria, as described in [28,29].

The number of users with patient roles and physiotherapist collaborators is increasing with the use and dissemination within the application. Patients can determine which level of exercises they need or are ready to perform inside the application. They can also watch videos that allow them to perform the exercises, interact with the chatbot to solve doubts, or contact the physiotherapists via chat or video conference. The physiotherapist users, when they join the application, can request validation so that they have access to the role of the physiotherapist. Once they have been granted the physiotherapist permission, they can guide the different patients who need help with the performance of exercises. Furthermore, each of the physiotherapists can keep track of their patients by recommending to each of them which exercises they should perform. They can also keep track of progress through the means of contacts that are allowed within the application.

Currently, in the application market, there are several methods available when launching an application to the market. These applications can be developed for a series of devices or a specific operating system, or we can focus the development on a technology that allows it to be used in most devices without having to carry out custom development for each type of device. For this reason, it has been determined to develop a progressive web app. The main characteristic of this technology is that we offer an application through the web with the same common web technologies such as HTML, CSS, and JavaScript, making the application compatible with any device that can render a web page, be it a computer, tablet, mobile, or even television with a web browser.

**Figure 2:**
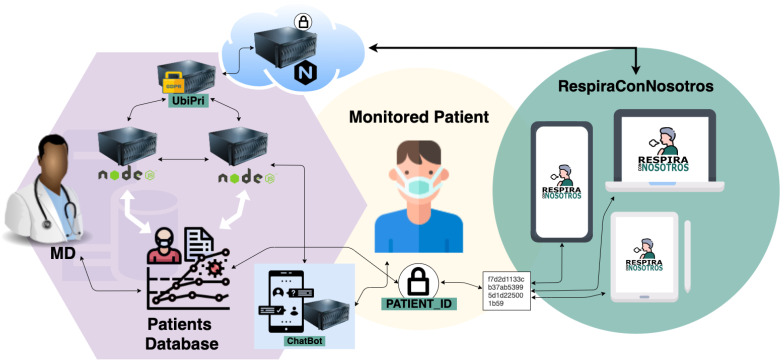
Architecture of rehabilitation application.

Within web development technologies, we have chosen VueJs [30], a web application development framework that is currently in fashion and allows for rapid and modular development, allowing the addition and integration of functionalities already developed by the community. On the server-side, use is made of a nonrelational MongoDB [31] database that allows simply modifying the data schemata with the integration of new functionalities. For the creation of the API that allows storage and obtains all the data of the application securely, NodeJS [32] is used. Apart from this, NodeJS also allows the creation of a WebSocket to carry out instant communications of the chat integrated into the application. Finally, Nginx [33] is used as a web application server and load balancer. Application deployment architecture can be seen in Figure 2.

In the application presented here, patient identification is stored as a unique ID, and managed by a data privacy middleware called UbiPri [34]. This middleware presents an implementation of algorithms that best fit the criteria, parameters, and information for handling data privacy based on patient history in the virtual environment.

**Figure 3:**
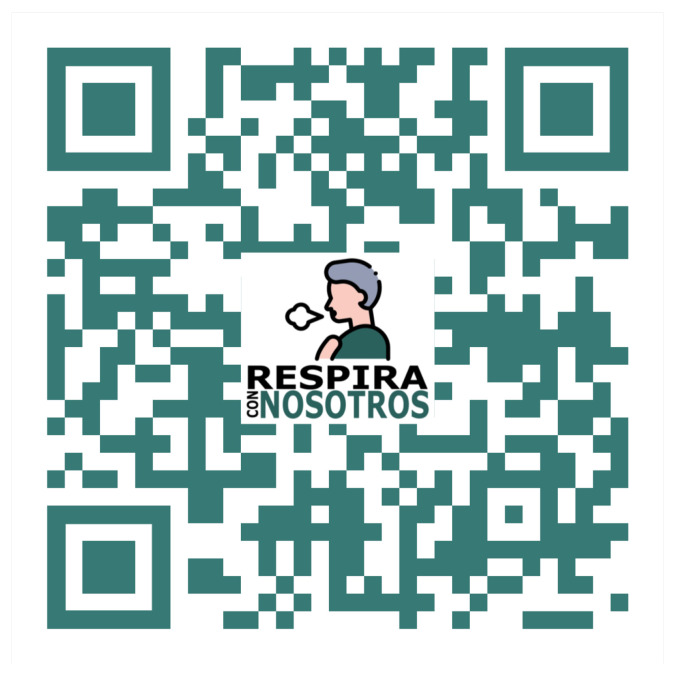
QR Code from the application.

Next, we proceed to a more detailed use explanation of the application. First, the application location; It can be done through the domain respiraconnosotros.es (accessed on 20 April 2021) or through a QR Code (Figure 3).

Through this link, the application will redirect users to the main page. A notice will advise if patients want to anchor a shortcut on our device. Initially, four tabs synthesize the information about the application; the first: an application brief description; the second: application objectives; the third indicates the expert contact possibility (specialized physiotherapist in this case) through a chat or video call, and the fourth, informs professionals who want to join to help patients.

The next step will be the registration to access. There are two types of registration, one for patient users and one for professional users. In both, users will be asked for a name, an email, a password, and password confirmation. The registration is also free.

Before describing each of the sections of the application, the functionality of the chatbot within the application will be explained in detail. The first interaction is realized by the user with the app; the user is invited to interact with the chatbot that will help him to determine which is the user’s starting level in the exercise performance. To determine the starting point level, the chatbot asks the user several questions to determine how they feel about doing daily tasks and thus determine the appropriate level.

Apart from this, we have added to the chatbot the extra functionality of reminding the user for every 3 days of inactivity within the application to perform the exercises daily to achieve the goals. In this case, the user is allowed to deactivate this option in case they do not want to be disturbed by this.

Each week, the bot will also ask the user a few questions to see if the user has improved from the previous week. These questions are configurable by the system managers.

Finally, the chatbot is also allowed to remind the user that the level of the exercises can be increased if the user can perform the repetitions of the exercises at the current level without fatigue.

**Figure 4:**
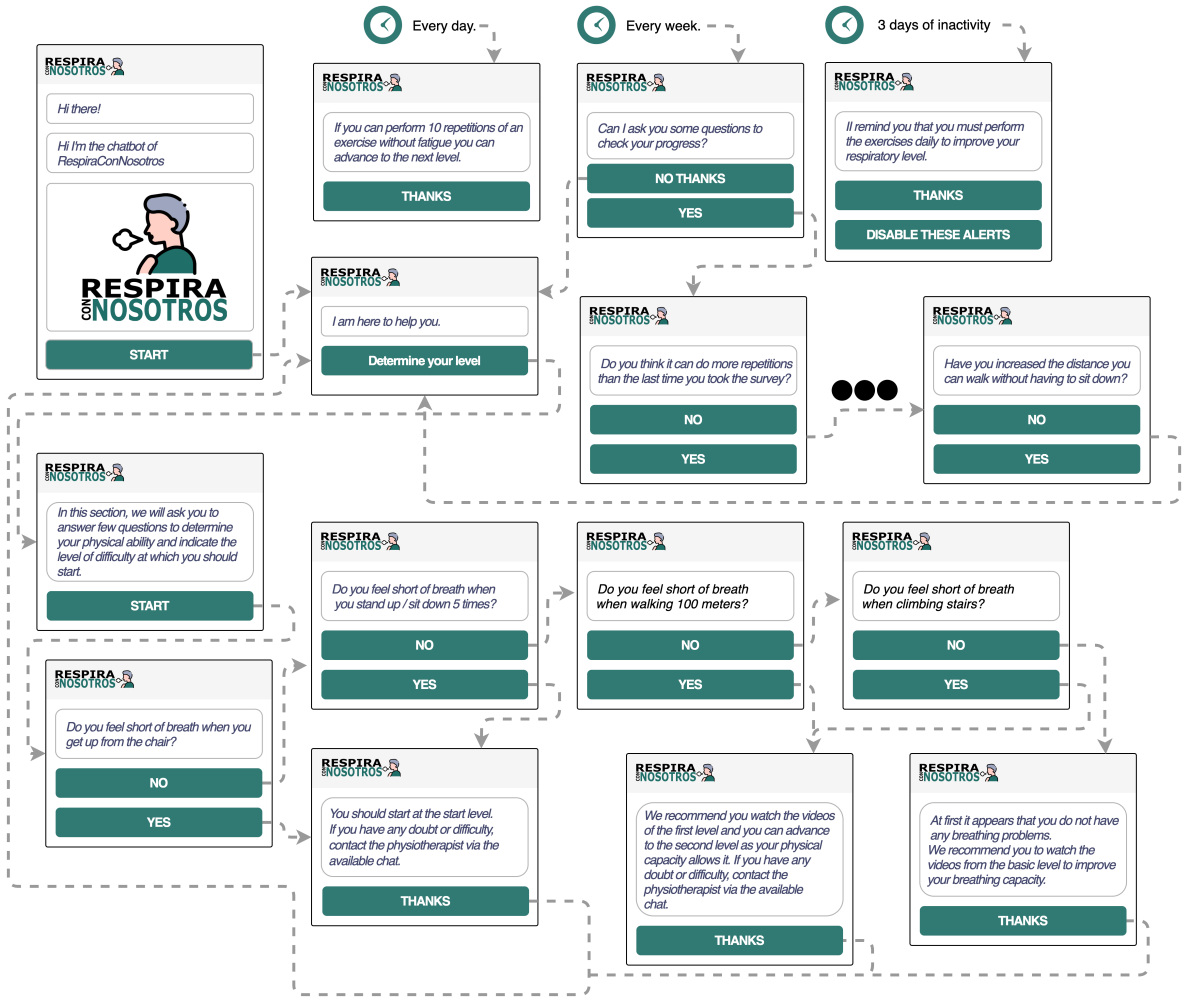
Chatbot iteractions.

The chatbot diagram can be seen in Figure 4.

The main page contains the videos and offers several options to move within the page. They are located at the bottom of the screen and five options appear: “Home”, “FAQ” (frequent answer questions), “Chat”, “Can I help?”, and “Exit” from the left to the right side.

**Figure 5:**
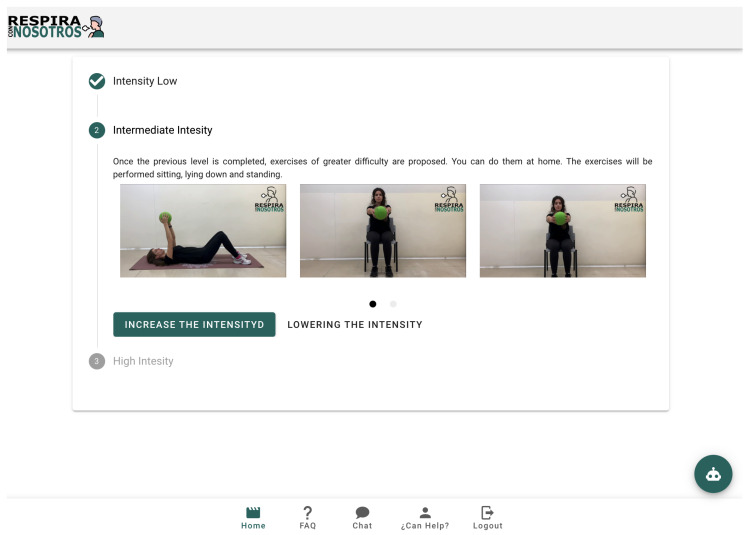
Home tab.

The first tab directs the user to the respiratory exercise section where we can observe three exercise levels in which we find a total of 20 respiratory exercises. In this exercise guide, the levels correspond to three difficulty levels (low, medium, and difficult) to allow personal adaptation to the personal patient functional status. By clicking on the video, it will start playing. All videos have an oral explanation during their implementation to improve their comprehension and reproduction by the patient. There is no limit to the reproduction number and the user can stop and review it as many times as they need to understand the exercise. Details can be seen in Figure 5.

The second tab at the bottom is the FAQ section; this is a space where some frequently asked questions have been included to solve common doubts. These questions are concerning the most suitable frequency to implement exercise, the number of repetitions they must do, or indications to select the appropriate level in which the patient should start their program. All of these questions or doubts have a short explanation to clarify and solve them.

**Figure 6:**
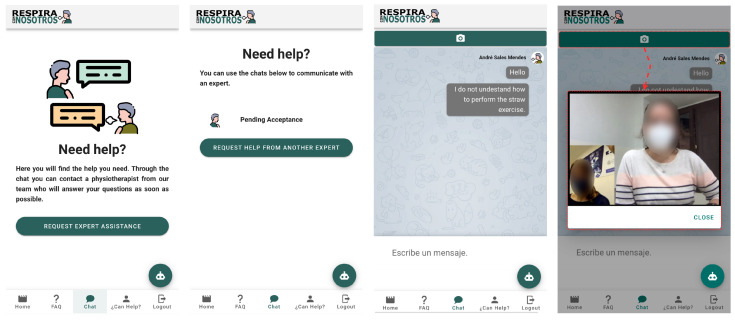
Chat tabs.

The third is the Chat tab. This space is aimed to ask questions with personalized attention through chat. Users could write their questions and a physiotherapist will solve the questions through this chat. A physiotherapist will solve your questions through this chat. If questions persist and the written communication is not enough, there is the video calling option where users could ask the professional and the physiotherapist can answer, explain, or show through the camera the exercise in which the patients have difficulties. These tabs are presented in Figure 6.

**Figure 7:**
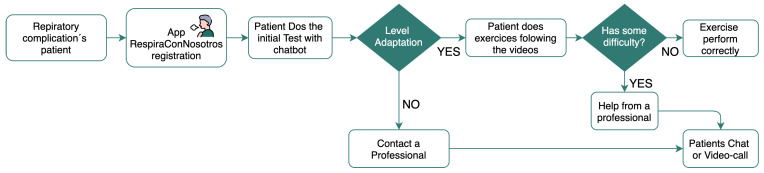
Diagram flow.

Finally, the fifth section of the bottom line corresponds to “exit” to leave the application. In Figure 7, a flow diagram about the complete procedure is shown.

There is a considerable number of patients that need these services; to expand service coverage to help as many patients as possible, and to offer the chance to collaborate in these treatments, professional registration was created. At first, the same personal data will be required as in the case of user registration: name, email, password, and password confirmation. Once located on the main page, we will click on the fourth tab. Professionals will be redirected to a screen whose title is “Do you think you can help?” with a short description. To complete the registration, professionals must click on the green box: “Start helping”.

**Figure 8:**
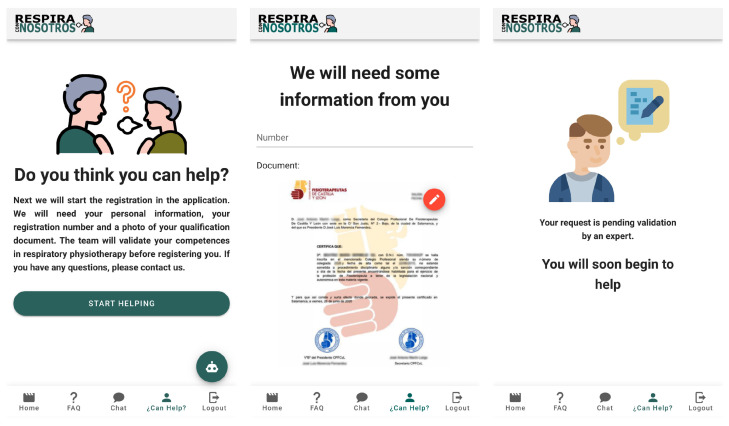
Professional registration.

At this time, a membership number and a supporting document will be requested to justify their training. Health is an important issue because an official training certificate will be required to be admitted. Our team will validate the request and if it is correct, it will be registered in the system to begin the collaboration (Figure 8).

As a registered professional, you will receive notification when there is a pending request and he must accept it to contact the user. Initially, it will do through the chat available, but he can also make a video call. The application diffusion will be carried out by several sources. The health system will be contacted through medical consultations and with posters; in the same way, it will be offered to COVID-19 patients and respiratory diseases associations (ASACOVID, FENAER, SEPAR). Outreach campaigns are carried out on the media and social networks.

### 2.3. Patients Recruitment and Exercise Prescription

The recruitment of volunteers was carried out by physiotherapists, who offered the program to patients who had suffered from COVID-19 reported complaints of fatigue and dyspnea. The volunteers were evaluated by the physiotherapists using the Borg Scale tool, widely used to know the level of referred effort [35,36]. To this end, the volunteers were asked to mark the effort perceived when climbing on floor stairs at the beginning of the program and the end of it. The Borg scale is really useful to measure exercise intensity and cardio-respiratory function [36]. Regarding the Borg scale results, volunteers’ functional level was assessed and the physiotherapists made proper recommendations.

To evaluate viability, volunteers and physiotherapist were asked to complete a satisfactory questionnaire (it has been included as Appendix A).

## 3. Results

The tool was created and previously tested in healthy subjects to determine the type of exercise and their adaptations for respiratory patients, to correct potential bias, and to test the viability of the application. After that, a pilot study with post-COVID-19 patients was implemented to test it. All the patients voluntarily agreed to carry out the study and were duly informed about the objectives and development of the study. This process was carried out following the Helsinki Declaration of 1964, Ethical Principles for Medical Research Involving Human Subjects. A total of 15 subjects participated in the study, eight men (between the ages of 28 and 38) and seven women (between 29 and 37 years old). The study was carried out for a month; at the beginning, the intervention guidelines were explained to the participants, which consisted of performing the exercises three times a week, with a duration of 20 min each time. They were instructed on how to enter the application and navigate its contents. They were explained that they should start with easy-level exercises and progress according to their abilities, without feeling fatigued after performing any of the exercises. Communication with physiotherapists was also explained and was carried out thanks to the collaboration of four expert physiotherapists in the field available to solve any doubts they could have during the exercise implementation.

After carrying out the intervention for a month, in which the participants performed the exercises at their homes and contacted the collaborating physiotherapists as many times as they believed necessary, a new evaluation of the intervention was carried out. To attempt our aim, a survey was conducted to evaluate the satisfaction and the effect of the program on the participants. A satisfaction survey was also carried out for the collaborating physiotherapists to collect professionals feedback. Attending to participants’ answers/As for the participants, the majority had a good or very good connection to the platform (80%); only 20% valued the connection as normal. Only one of the participants found connecting problems, but they were solved by the platform’s technical team on the same day.

About exercise exertion, 47% of the participants considered the exercises as very easy to perform, 40% considered it easy, and the rest perceived medium ease. Regarding the material needed to carry out the exercises, the participants considered that it was accessible (53%) or very accessible (47%) material.

**Figure 9:**
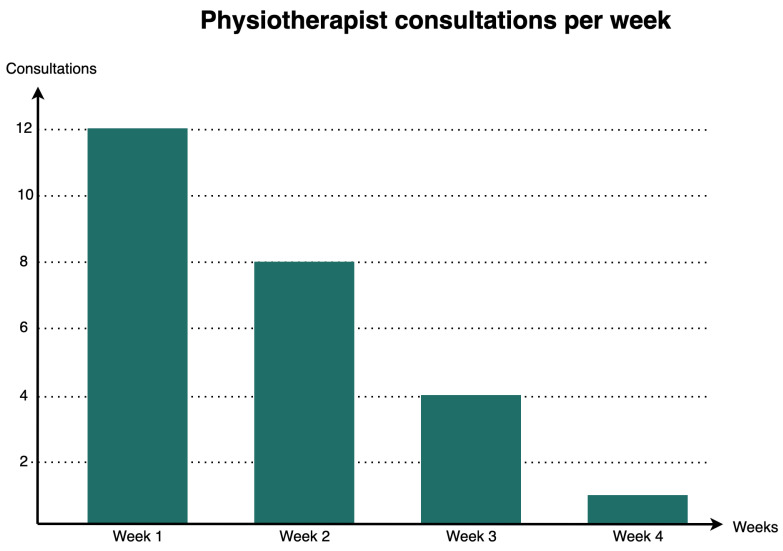
Physiotherapist consultations per week.

Most of the participants had no difficulty performing the exercises (67%) and the rest had very little difficulty. However, the vast majority contacted the collaborating physiotherapists (73%), and all of those who did so considered that their doubts had been solved. Most of the contacts were established during the first weeks and they declined as the intervention time progressed as we can see in Graph in Figure 9.

**Figure 10:**
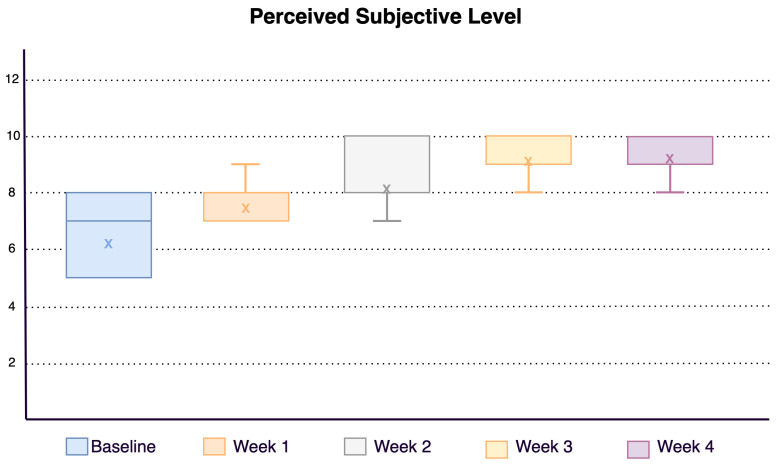
Perceived subjective level.

Regarding the effect of the program, it was clearly seen how the level of effort perceived subjectively by the participants was increasing, which implies that the respiratory function was better as the weeks progressed Graph in Figure 10. All participants improved by at least two points from baseline. Concerning functional assessment, all participants improved their score on the Borg scale after performing the intervention with the application.

All 15 participants agreed that they would recommend the application to family and friends in their situation, as they felt it was beneficial to their health. In the collaborating physiotherapists survey, it was determined that 75% of them had a very good connection and 25% good. 50% indicated the material used as accessible and the rest as very accessible. All pointed out the absence of technical problems during the intervention and the usefulness of the application in clinical practice.

Among the greatest advantages they pointed out are the accessibility to a greater number of patients, the possibility of contacting them during periods of isolation, the possibility of monitoring home-based programs, and the greater adherence that this can lead to. After conducting the pilot phase, it is completed with a fully functional and accessible application, with a high degree of satisfaction from the study subjects. Currently, it is in the clinical trial process with a randomized and controlled trial with 12 weeks duration, a frequency of three weekly sessions, and a follow-up at 3 months. This trial is registered with NCT number: NCT04703478. Patients with COVID-19 complications are invited to participate.

## 4. Discussion

Digital health care developing solutions have faced the COVID-19 pandemic situation. Application developing has been one of the most popular ways to connect people. Different applications have been implemented since the COVID-19 disrupt.

Some authors highlight the need to create mobile applications to assist in the symptoms self-assessment compatible with COVID-19 as well as for the notification of hospitalizations to control and combat the pandemic [16]. It should be noted that most of the applications created during the pandemic were developed by government entities. Regarding the purposes, general information about COVID-19, collection of symptoms, and follow-up of contacts predominated [37].

It seems clear that the close contacts control contributes to the control of the COVID spread. Applications based on contact tracing seem to have good acceptance in European countries where the willingness to install the application is high [38]. However, protecting citizens’ privacy guarantees is necessary, which is why ethical guidelines are necessary to regulate these applications that process sensitive health-related data [39].

The development of applications for pandemic control and case tracking continues to be a basic need [16]. Telemedicine has advanced throughout the pandemic through tele-education, multidisciplinary groups creation and medical equipment, psychological telecare, prescription and distribution of drugs, and telemedical care through telephone consultation [40]. The creation of applications that allow a safe treatments for patients as well as an optimization of resources from health professionals are part of the ethical approach from health in order to reach a greater number of users and the community benefit. Applications monitoring physical rehabilitation [41] are focused on combating the possible consequences or complications of COVID-19 or other issues, as improving the lung capacity of all users who wish to use it from the safe environment of e-health, even at home.

Yerrakalva et al. [42], in their recently published article, investigated the interventions effects with mobile applications on sedentary lifestyle and physical activity in older people. These applications have shown great potential to promote changes in health status and better health in users who used them compared to those who did not [3]. In this sense, mobile applications to promote healthy style changes have been part of our lives for years; however, there is no guide to help the user on which application is more convenient or applications regulation focused on health [5]. Point that in our application is solved since it has been created by health experts who have developed the content specifically for this purpose. Furthermore, user satisfaction and the perception of improvement reinforce and support this argument.

In other applications for patients health monitoring, specifically cancer patients, different scenarios have been used, such as screening patients and preparing the face-to-face visit in which the measures relating to the prevention of transmission are reported as well such as remote monitoring scenarios and even monitoring in the hospital [43,44]. The possibilities of RespiraConNosotros application are not limited to home environment, it can be used in the hospital environment and in the follow-up to hospital discharge.

As different authors point out, pulmonary rehabilitation is the nucleus of medical rehabilitation in COVID patients. Different respiratory exercise work must be done depending on symptoms intensity and period of illness; on these way, patients are rate on outpatient mild disease, acute inpatients, and post-acute patients [45]. All of them could benefit from the use of RepiraConNosotros application following the physiotherapist instructions because of the gradation that the exercises selected have from low to high intensity.

RespiraConNosotros application is a viable and important guide to patients who have suffered COVID disease to recover their respiratory function complications. Patients with respiratory sequelae, especially these COVID patients who need professional supervision of their treatments, to indicate and graduate the exercises that patients must do to achieve the greatest functional recovery could improve their health with this application. There is no access limitation by sex, age, culture, or cultural level. All people with access to a mobile phone and an Internet connection will be able to benefit from this technology; it can also be integrated with other devices and Internet [46].

The application potential is enormous since it is available for all platforms (PC, Android, iOS), it has a simple use, and it does not require installation. It is accessible to the population and is a good complement to face-to-face therapies to the extent that they can be performed again. However, it is not intended to replace any therapy. One of the points pending attention in this work is about performance and scalability in cloud computing since the application uses video calls for patient care. For this, we should follow a bandwidth reduction model as presented in [47], which shows in experiments that it can reduce execution time by up to 77% and improve CPU efficiency by up to 49%.

Furthermore, the exercises can always be supervised and the patients/user can contact a physiotherapist who will guide them during the exercise, their evolution, or for the resolution of any doubts that may arise. Among the possible limitations that we can mention are the limited number of exercises, only Spanish language and the access impediment to visual impairment people.

## 5. Conclusions

In this article, the development, implementation, and viability of a home-based telerehabilitation system for respiratory patients is presented. The development is based on a Progressive Web App, with the same common web technologies (HTML, CSS, and JavaScript) making the application compatible with any device that can render a web page. The respiratory rehabilitation exercise has been designed by experts based on clinical guides and evidence. Important health and safety recommendations are included, guiding the users through their progress.

It is important to clarify that users are always supervised and guided by physiotherapists. The audiovisual content has been created by health care experts, becoming a great complement to previously prescribed therapies. Additionally, chat and private video call are available.

As health care providers have been one of the most affected groups by the COVID-19 and the rehabilitation programs have been stopped, the RespiraConNosotros application is the newest tool for physicians giving the safest solution to continue treatments. However, its applicability is not only reduced during quarantine periods; it is also a good asset to improve adherence and motivation in traditional rehabilitation programs (as a complement or patients follow-up).

Comparing this application with similar ones, the contribution becomes clear. Most applications developed to face the COVID-19 situation are based on contact tracing and health information. Our project gives a viable solution to a great current problem, the incoming number of people who need to improve their respiratory performance due to the COVID-19 situation.

As a final conclusion, It can be highlighted that RespiraConNosotros application is a simple, viable, and safe alternative for the improvement and maintenance of respiratory capacity and patient’s functionality affected by SARS-CoV-2.

In future lines, full implementation of the app is planned with more language integration to favor access worldwide. Furthermore, a greater respiratory exercise variety ranked by category is considered introduced, as well as other therapies addiction (such as stretching exercises or strength exercises among others). Considering functional diversity adaptation, subtitles and voice commands are required to ensure accessibility. This line of investigation will be developed under the registry NCT04703478 and following the ethical considerations established.

The performance and scalability of the application are points to be improved with new variables, in addition to those already used in the related work and the UbiPri middleware platform used.

## Figures and Tables

**Figure A1:**
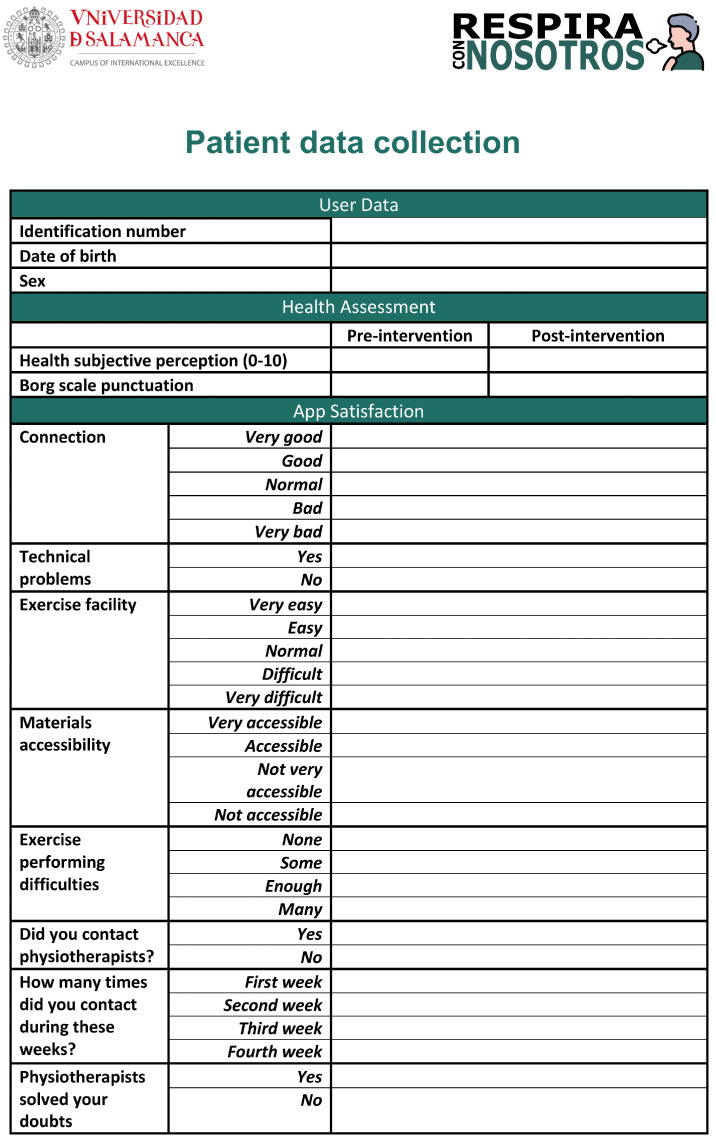
Patient data collection survey.

**Figure A2:**
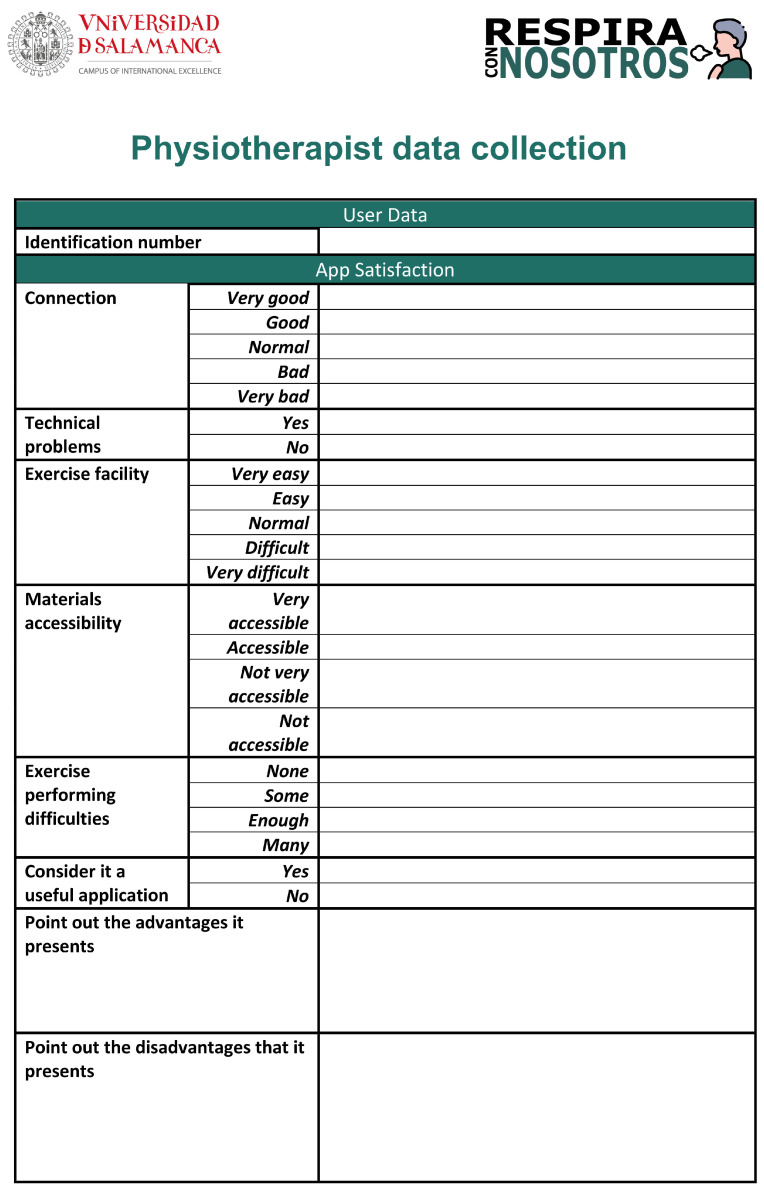
Physiotherapist data collection survey.

## Data Availability

The data presented in this study are available on request from the corresponding author. The data are not publicly available due to this research is ongoing.

## Abbreviations

The following abbreviations are used in this manuscript:

ASACOVID	Asociación Afectados por Coronavirus
API	Application Programming Interface
COPD	Chronic Obstructive Pulmonary Disease
CSS	Cascading Style Sheets
FAQ	Frequent Answer Questions
FENAER	Federación Española de Asociaciones de Pacientes Alérgicos
	y con Enfermedades Respiratorias
GDPR	General Data Protection Regulation
HIPAA	Health Insurance Portability and Accountability Act
HTML	Hyper Text Markup Language

HIV	Human Inmunodeficiency Virus
iOS	iPhone Operative System
NCT	Number of Clinical Trial
PC	Personal Computer
SARS-CoV-2	Severe Acute Respiratory Syndrome—Coronavirus
SEPAR	Sociedad Española de Neumonología y Cirugía Torácica
